# An Account of the Good Effects of Opium in the Case of a Person Poisoned by Digitalis

**Published:** 1794

**Authors:** Thomas Beddoes


### II. *An Account of the good Effects of Opium in the Case of a Person poisoned by Digitalis. Communicated in a Letter to Dr.* Simmons, *by* Thomas Beddoes, *M. D.*

THE following case may perhaps be instructive to practitioners the first time they meet with a difficulty of the same kind.

A person, much emaciated and enfeebled, and labouring under anasarca and hydrothorax, took, by mistake, from two to four doses of an infusion of digitalis, more than were ordered for him. He had nausea during a considerable part of Tuesday night, which, by 10 o'clock on Wednesday morning, had increased so much, that every five or ten minutes he threw up a small quantity of bile, with the most dreadful retchings that can be imagined.

I had once, while I was a student of medicine, seen a much stronger patient destroyed by digitalis; but the practice of the physician who had the management of the case was exceedingly feeble and fluctuating. He only adminiftered fome flight opiates, a little port wine, and effervefcing draughts.

From the weaknefs of my patient, and the terrible effects always produced by digitalis, when improperly adminiftered, I entertained very flight hopes of his recovery. I refolved, however, to attempt fomething towards his relief. For a fhort time I hefitated between opium and brifk emetics, (of white vitriol and muftard feed, for inftance) which laft, I hoped, might change the action of the ftomach and liver, induced by the digitalis. I determined in favour of opium, of which I ordered three grains to be given at two dofes; one immediately, and the other at the end of an hour; and afterwards fifteen drops of tincture of opium, every hour, in port wine, till the patient fhould fall afleep.

He dofed a good deal in the evening, and the vomitings had become lefs frequent by Thurfday morning, occurring never oftener than once in half an hour, and fometimes only every hour and a half. The patient flept between each fit of ficknefs, and always awaked with naufea.

I gave him now fixty drops of tinct. opii by clyfter, and three dofes of 8 grains of pulv. Ipecac. comp. made into pills with extract. cicut.

to be taken at the interval of two hours between each dofe, and ordered the clyfter to be repeated in the evening.

During the night he perfpired copioufly, always awaking fick; the fickneffes more infrequent, but fometimes attended with fingultus.

On Friday he ceafed to vomit bile; and as he feemed entirely under the influence of opium, no medicines were prefcribed on that day.

Saturday—he had drank toaft and water during the night, which agreed well with his ftomach. He had no ficknefs this day; he had been able to lie down ever fince Wednefday, which he had been utterly unable to do before, and his feet only fwelled a little towards night. He now began to eat with a very keen appetite, and drank almoft half a bottle of wine a day; he had before led a very abftemious life. The bark in fubftance, with aromatics, was ordered for him, and he took about half an ounce every other day, for four times. The fwelling of his legs, towards night, went off, and he has now, for fome time, been perfectly well.

This cafe fhows that opiates may be freely adminiftered to a perfon poifoned by digitalis. I dare not draw any bolder conclufion from a fingle cafe, but I would purfue the fame plan of treatment under a fimilar emergency.

P.S. The pulfe did not come down below fixty; perhaps the opium counteracted the effect of the digitalis.

*Briftol Hot Wells,*

*July* 14, 1793.

